# Environmental degradation, eco-anxiety and post-traumatic stress symptoms among Palestinian adults: The mediating role of coping strategies

**DOI:** 10.1017/gmh.2024.40

**Published:** 2024-10-08

**Authors:** Dana Bdier, Guido Veronese, Fayez Mahamid

**Affiliations:** 1 An-Najah National University, Nablus, Palestine; 2 University of Milano-Bicocca, Milan, Italy

**Keywords:** environmental degradation, eco-anxiety, post-traumatic stress symptoms, coping strategies, Palestine

## Abstract

The current study aimed to test the association between environmental degradation, eco-anxiety and post-traumatic symptoms, and whether coping strategies mediate the association between these variables among a sample of Palestinian adults. The sample of our study consisted of 554 Palestinian adults, of whom 392 identified as female and 162 as male. Participants’ age ranged from 19 to 54 years old (M = 35.8, SD = 12.31). They were all recruited from online advertisements, e-mail campaigns and social media. The findings of our study revealed that post-traumatic stress symptoms positively correlated with environmental degradation, eco-anxiety and avoidant coping and negatively correlated with problem-focused coping and emotion-focused coping. Results of structural equation modeling revealed that coping strategies mediated the association between environmental degradation, echo-anxiety and post-traumatic stress symptoms. The findings of our study emphasize the need for tailored psychological support and coping strategies for individuals experiencing eco-anxiety and post-traumatic stress symptoms in the face of environmental challenges.

## Impact statement

Environmental degradation and eco-anxiety are considered global issues, but the condition can worsen in Palestine due to ongoing military occupation with its policies and practices. Environmental degradations as a consequence of the Israeli occupation require strengthening mental health interventions targeting Palestinian people suffering trauma, and mental health-related problems. The mission of formal and popular mental health interventions (institutionalized and non-institutionalized) should be focused on promoting positive coping strategies and resilience among Palestinians to effectively deal with continuous traumatic situations.

## Introduction

Environmental degradation encompasses the deterioration of environmental resources, including air, water and soil, resulting in the degradation of ecosystems and the loss of biodiversity (Chopra, 2016). The current global scenario is marked by environmental degradation driven by a range of ecological challenges such as air and water pollution, and climate change, with human activities predominantly implicated as the primary causative agents of this environmental decline (Tyagi et al., 2014; Chandra, 2015).

In the Palestinian territories, the environment has witnessed significant deterioration due to actions stemming from Israeli military occupation. These actions encompass resource depletion, most notably through the establishment of settlements, which exert detrimental effects on the Palestinian environment. Additionally, land confiscation and the restriction of Palestinian citizens from accessing their lands for agricultural purposes, the depletion of Palestinian water resources, wastewater pollution, solid waste accumulation, air pollution and noise pollution, along with the destruction of cultural heritage and the agricultural sector, all contribute to this environmental crisis (Bdier et al., 2022; Veronese et al., 2022).

The vulnerability of communities with limited resources to mitigate the adverse impacts of the climate crisis is expected to increase the prevalence of mental health issues, as demonstrated by Clayton et al. (2017). Furthermore, the failure of Palestine to achieve any of the Sustainable Development Goals (SDGs), as outlined in the 2019 report of the Middle East/North Africa (MENA) region, places Palestinians at heightened risk of experiencing eco-anxiety (Lambert et al., 2020).

Environmental conditions such as climate change, environmental degradation and pollution are now recognized as some of the most profound global health threats in the present century (Coffey et al., 2021). Notably, the American Psychological Association (APA) predicted climate change to emerge as the most significant challenge to human well-being (APA, 2009). The World Health Organization (WHO) also anticipates climate change to cause 250,000 annual deaths between 2030 and 2050 due to malnutrition, malaria, diarrhea and heat stress (WHO, 2021). Moreover, environmental degradation can be linked to increased poverty, species loss, famine, acute and chronic medical conditions, overcrowding, climate variations and conflicts, thereby infringing upon human rights (Tyagi et al., 2014).

Consequently, there has been a growing interest in environmental and ecological issues and their impact on health and mental well-being. Various terms, such as eco-anxiety, eco-depression, eco-guilt and solastalgia, have emerged in news, social media and blogs to describe the mental health responses to ecological crises (Pihkala, 2020; Hogg et al., 2021; Stanley et al., 2021).

While no universally accepted definition of eco-anxiety exists, Panu’s study (2020) suggests several commonly used definitions: (1) chronic fear of environmental catastrophe, (2) the generalized belief in the impending collapse of ecological foundations of existence and (3) nonspecific anxiety about our relationship with the natural environment (p. 4). Eco-anxiety manifests in various ways, with individuals experiencing panic attacks, feelings of despair and grief and some even choosing not to have children due to uncertainties about the future quality of life (Arcanjo, 2019). However, eco-anxiety shares common features with anxiety disorders, as individuals with eco-anxiety exhibit symptoms including fear, anger, exhaustion, powerlessness, feelings of loss, helplessness, phobias, panic attacks and insomnia (Baudon and Jachens, 2021).

Eco-anxiety can result from direct or indirect exposure to ecological or environmental traumatic events, such as earthquakes, hurricanes, land loss, pollution and floods, or through awareness of anticipated environmental threats in the future (Wang et al., 2023).

Studies have shown varying prevalence rates of eco-anxiety. For example, a study in Australia found that 9.37% of participants reported significant eco-anxiety (Patrick et al., 2022). In the USA in 2018, 70% of individuals expressed worry about climate change, with 51% feeling helpless (Jain and Jain, 2022). In 2020, over 57% of English children and young adults experienced distress regarding climate change (Jain and Jain, 2022). A study in the Arab World found differences in climate-related mental health issues among different countries, with some experiencing more fear, anxiety, alienation and somatic symptoms, while others exhibited higher rates of climate depression (Arnout, 2023).

Environmental degradation has also been linked to individual mental health issues. Research has revealed a negative relationship between concern about ozone pollution and individual well-being, as well as a positive relationship between concern about species extinction and well-being (Ferrer-i-Carbonell and Gowdy, 2007). The absence of green spaces and environmental degradation are significant risk factors for poor mental health (Wang et al., 2021). Changes in landscapes and climate have been associated with increased depression, anxiety, stress and grief (Doherty and Clayton, 2011), and environmental degradation has been negatively associated with health and mental health, leading to stress, anxiety and depression among Palestinian adults (Bdier et al., 2022).

In the context of environmental catastrophes, mental health can be directly impacted, potentially resulting in post-traumatic stress disorder (PTSD). Individuals exposed to life-threatening and traumatic events are at higher risk of developing PTSD, characterized by symptoms like flashbacks, heightened arousal and avoidance of reminders of the traumatic event (Padhy et al., 2015).

Across the Palestinian territories, eco-anxiety and environmental degradation have had a negative impact on residents’ physical and mental health; many, including women and children, suffer from anxiety, distress depression and psychological trauma (Marie et al., 2016; Mahamid et al., 2023). Bdier et al. (2022) tested the association between environmental concerns and mental health distress among Palestinians; the findings revealed that environmental concerns and eco-anxiety were positively correlated with mental health distress among Palestinians. Mahamid et al. (2024) explored environmental concerns and challenges among university students in geopolitically risked Palestine; the findings showed that there are several environmental concerns and challenges faced by Palestinian university students that negatively affect mental health issues, such as environmental deterioration, military occupation and human rights violation.

Various coping strategies are employed to address psychological distress related to environmental degradation and eco-anxiety. These strategies can be categorized as active coping (functional) or avoidant coping (dysfunctional) (Al-Dubai et al., 2011). Active coping involves addressing the problem directly through behavioral and cognitive efforts, while avoidant coping focuses on managing emotions by avoiding thoughts related to the source of distress, often through denial, distraction or substance use (Gustems-Carnicer et al., 2019; MacIntyre et al., 2020).

In the face of environmental degradation and eco-anxiety, hope and communal support emerge as effective coping strategies. Hope can motivate individuals to confront environmental challenges, while communal support fosters mutual assistance and a sense of efficacy (Ojala, 2018). Community connections are also pivotal in coping with eco-anxiety in the context of climate change and environmental degradation, as they facilitate information-sharing and mutual support (Kelly, 2017). Additionally, coping self-efficacy, hope, social support and optimism about the future are strong predictors of positive mental health outcomes in the face of weather-related disasters and environmental conditions (Trombley et al., 2017).

## Current study

Environmental degradation in Palestine is a significant and multifaceted issue that has been exacerbated by various factors, including conflict, occupation, population growth and resource scarcity. Regarding resource depletion, the Israeli–Palestinian conflict has had a detrimental impact on the natural resources in the region. This includes the depletion of water resources, destruction of arable land and deforestation. Palestinians face challenges in accessing and managing essential resources. Water scarcity is a critical issue in Palestine. Palestinians face limited access to clean and reliable water sources. The overextraction of groundwater, often in violation of international agreements, has led to the salinization of water sources and the drying up of wells. Environmental pollution is a growing concern. Pollution from various sources, including industrial facilities and untreated sewage, affects air and water quality. The lack of proper waste management systems contributes to pollution issues. Land confiscation because of the construction of Israeli settlements in the West Bank has led to the confiscation of Palestinian land, which often results in the destruction of agricultural areas and natural habitats. These settlements can also contribute to pollution and resource scarcity. Deforestation is a problem in Palestine, primarily due to the demand for wood and agricultural expansion. This has adverse effects on local ecosystems, contributing to soil erosion and loss of biodiversity. Air pollution is a concern in urban areas, particularly in densely populated regions. The use of fossil fuels and a lack of environmental regulations contribute to poor air quality. Loss of biodiversity due to the destruction of natural habitats and ecosystems has led to a loss of biodiversity in the region. Many species of plants and animals are endangered or have become extinct due to habitat destruction.

Further, environmental degradation has direct implications for public health. Poor water quality, air pollution and inadequate waste disposal systems can lead to various health problems among the Palestinian population. Finally, the climate change is exacerbating existing environmental challenges. Rising temperatures, changing precipitation patterns and increased frequency of extreme weather events pose additional threats to Palestinian agriculture and infrastructure.

Thus, the political and economic challenges faced by Palestinians often limit their ability to implement effective environmental mitigation and conservation measures. Efforts to address environmental degradation in Palestine face numerous obstacles, including political instability and limited resources. International organizations and NGOs have been involved in projects aimed at improving environmental sustainability in the region. However, a comprehensive solution to environmental degradation in Palestine would require addressing the broader political and socioeconomic issues contributing to the problem.

The present study seeks to investigate the intricate relationships between environmental degradation, eco-anxiety and post-traumatic symptoms (PTSs) within the context of Palestinian adults, with a particular focus on the potential mediating role of coping strategies. Building upon the extensive body of prior research (Ferrer-i-Carbonell and Gowdy, 2007; Doherty and Clayton, 2011; Kelly, 2017; Rossati, 2017; Trombley et al., 2017; Ojala, 2018; Cianconi et al., 2020; Palinkas and Wong, 2020; Wang et al., 2021; Bdier et al., 2022), this study formulates three distinct hypotheses to guide its investigation:

Hypothesis 1:
Environmental degradation is expected to exhibit a positive association with both eco-anxiety and PTSD among Palestinian adults. This hypothesis draws upon previous findings indicating the adverse impact of environmental degradation on mental health (Cianconi et al., 2020; Bdier et al., 2022). It posits that as environmental degradation intensifies, individuals may experience heightened eco-anxiety and an increased susceptibility to PTSSs, reflecting the distressing consequences of deteriorating environmental conditions on psychological well-being.

Hypothesis 2:
Coping strategies are expected to demonstrate a negative association with environmental degradation, eco-anxiety and PTSSs among Palestinian adults. Prior research suggests that effective coping strategies can mitigate the adverse mental health effects of environmental challenges (Doherty and Clayton, 2011; Trombley et al., 2017; Wang et al., 2021). This hypothesis posits that individuals who employ adaptive coping strategies are likely to exhibit lower levels of environmental degradation, reduced eco-anxiety and a decreased vulnerability to PTSSs.

Hypothesis 3:
Coping strategies are hypothesized to mediate the relationships between environmental degradation, eco-anxiety and PTSD among Palestinian adults. Building upon existing literature highlighting the protective role of coping strategies (Kelly, 2017; Rossati, 2017; Ojala, 2018; Palinkas and Wong, 2020), this hypothesis suggests that the effectiveness of coping strategies in buffering the impact of environmental degradation on eco-anxiety and PTSD will vary among individuals. Specifically, it posits that individuals with strong coping skills may experience weaker associations between environmental degradation and both eco-anxiety and PTSD, indicating a dampening effect of these coping strategies on the negative mental health consequences of environmental degradation.

In summary, this study adopts a comprehensive approach to assess the relationships between environmental degradation, eco-anxiety and PTSSs among Palestinian adults, while also exploring the potential moderating influence of coping strategies. These hypotheses are grounded in prior empirical evidence, offering a structured framework for the investigation of these complex interrelationships within the specific context of the Palestinian population.

## Methods

### Participants and procedures

The research was conducted in July 2023, targeting Palestinian residents of the West Bank of Palestine. Participant recruitment was executed through a multifaceted approach, encompassing online advertisements, e-mail outreach campaigns and utilization of social media platforms. The study’s objectives were conveyed through online channels, and individuals expressing interest in participation initiated contact by submitting an e-mail indicating their intent to partake in the research. Subsequently, all prospective participants were furnished with a comprehensive letter elucidating the study’s objectives and ethical considerations. A pivotal component of the recruitment process entailed the acquisition of written informed consent from each participant, signifying their unequivocal agreement to the stipulated conditions of study participation. In total, the study comprised 554 adult participants, of whom 392 identified as female and 162 as male. Geographically, 32.1% of participants hailed from villages and 63.5% from urban centers, while the remaining 4.3% originated from Palestinian internally displaced camps. In terms of educational attainment, 34.7% held a master’s degree, 54.9% possessed a bachelor’s degree and 4.3% held a high school diploma. Eligibility criteria for inclusion in the study were defined as follows: (1) participants had to be native Arabic speakers, (2) identify as Palestinians, (3) and maintain residency within the occupied Palestinian territories (oPt). Ethical clearance for the research project was obtained from the An-Najah Institutional Review Board (IRB) prior to commencing data collection. This adherence to ethical standards underscores the study’s commitment to safeguarding the rights and well-being of the participants and ensuring the integrity of the research process.

### Measures

The *Hogg Eco-Anxiety Scale (HEAS-16)*, developed by Hogg et al. (2021), was employed in this study to assess eco-anxiety. This self-report instrument consists of 16 items and is designed to measure affective symptoms of eco-anxiety, ruminative thoughts related to environmental concerns (e.g., “Unable to stop thinking about future climate change and other global environmental problems”), impairment in behavioral and social functioning (e.g., “Difficulty working and/or studying”) and anxiety concerning one’s impact on the planet (e.g., “Feeling anxious about the impact of your personal behaviors on the earth”). Participants rated their eco-anxiety on a 5-point frequency scale (1 = not at all and 3 = nearly every day). The internal consistency of the HEAS-16, as assessed through reliability analysis, demonstrated a high degree of reliability in evaluating the eco-anxiety of Palestinians (α = .87).

The *Environmental Appraisal Inventory (EAI)*, a 31-item instrument developed by Fridgen (1994), was utilized to assess various facets of environmental degradation. Participants were required to rate environmental hazards (e.g., water pollution, noise pollution, global warming and depletion of landfill space) across multiple dimensions, including the perceived threat of these hazards to themselves (Threat), the likelihood of being impacted by them (Affected), the likelihood of these hazards becoming problematic in their lifetime (Temporal risk), the likelihood of these hazards posing a problem in their local area (Spatial risk) and their knowledge of behaviors to mitigate the problems posed by these hazards (“Knowledge”). Ratings were made on fully labeled 5-point scales (e.g., Threat = 1 to 5 = extreme threat). The EAI exhibited a high level of reliability in evaluating environmental degradation among Palestinians (α = .88).

The *Impact of the Event Scale-Revised (IES-R)*, developed by Weiss and Marmar (1997), was employed to assess post-traumatic stress symptoms in response to traumatic and stressful events. The IES-R includes three subscales representing the main symptoms of traumatic events: hyperarousal, avoidance and numbing and intrusion. The hyperarousal subscale assesses anger, irritability and concentration difficulties related to traumatic experiences, while the avoidance and numbing scale evaluates avoidance of people, places and things associated with traumatic events. The intrusion subscale measures nightmares, distressing thoughts and intense emotions related to traumatic experiences.

The *Coping Orientation to Problems Experienced Inventory (Brief-COPE)*, developed by Carver (1997), comprises 28 self-report items designed to assess both situational coping strategies (how individuals cope with specific stressful events) and dispositional coping strategies (how individuals typically cope with stress in everyday life). This instrument encompasses three main coping strategies: Behavioral coping, emotional coping and avoidant coping, with 14 coping subscales, each containing two items. Respondents rate their use of coping responses on a four-point Likert scale (1 = I usually do not do this at all; 2 = I usually do this a little bit; 3 = I usually do this a medium amount; 4 = I usually do this a lot).

### Data analysis

**Figure 1.**
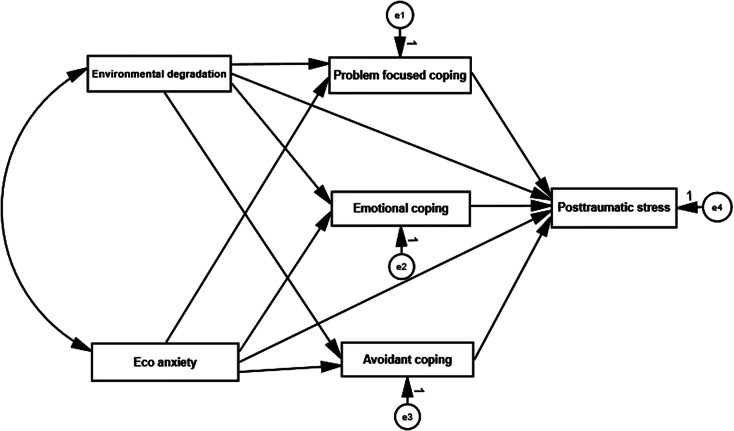
The conceptualized effect of environmental degradation and eco-anxiety on post-traumatic stress, and the mediating roles of coping strategies.

Descriptive statistics were employed to characterize the main features of the study variables. Additionally, correlations between study variables, including eco-anxiety, environmental degradation, behavioral coping, emotional coping, avoidant coping and PTSSs, were examined. Structural equation modeling (SEM) was utilized to assess the conceptual model depicted (see Figure 1). In this model, eco-anxiety and environmental degradation served as predictive variables, while behavioral coping, emotional coping and avoidant coping acted as mediating variables, ultimately influencing post-traumatic stress symptoms as an outcome variable. The SEM analysis demonstrated excellent fit indicators, including a Comparative Fit Index (CFI) of .97, Standardized Root Mean Square Residual (SRMR) of .04 and Root Mean Square Error of Approximation (RMSEA) of .03. The SEM model was analyzed using AMOS 25 statistical analysis software.

## Results

Descriptive statistics (see Table 1) for post-traumatic stress, environmental degradation, eco-anxiety, problem-focused coping, emotion-focused coping and avoidant-focused coping were tested as shown in Table 1. Environmental degradation yielded remarkable scores among participants, whereas post-traumatic stress symptoms, eco-anxiety, problem-focused coping and emotion-focused coping were rated moderately. Furthermore, participants revealed low scores for avoidant coping mechanisms. Moreover, all measures employed in this study demonstrated a high degree of reliability, spanning from .88 (eco-anxiety) to .93 (post-traumatic stress symptoms).

**Table 1.** Descriptive statistics for research variables (*N* = 554)

Variable	Mean	SD	Min	Max	Range	Skewness	Kurtosis	Reliability
Post-traumatic stress	2.79	.85	.17	5.00	4.83	.50	.520	.93
Environmental degradation	3.73	.89	.45	5.00	4.55	−1.37	2.423	.91
Eco-anxiety	2.90	.83	.69	4.92	4.23	−.31	−.146	.88
Problem-focused coping	2.79	.65	1.00	4.00	3.00	−.73	.190	.89
Emotion-focused coping	2.68	.48	1.33	3.75	2.42	−.46	.689	.93
Avoidant-focused coping	2.19	.56	1.00	4.00	3.00	.42	.254	.90

Results of the correlational analysis (see Table 2) show that post-traumatic stress symptoms positively correlated with environmental degradation (*r* = .11*, p* < .05), eco-anxiety (*r* = .64, *p* < .01) and avoidant coping (*r* = .52, *p < .*01) and negatively correlated with problem-focused coping (*r* = −.17, *p* < .01) and emotion-focused coping (*r* = −.19, *p* < .05). Environmental degradation positively correlated with eco-anxiety (*r* = .14, *p* < .05) and avoidant coping (*r* = .40, *p* < .01) and negatively correlated with problem-focused coping (*r* = −.15, *p* < .01) and emotion-focused coping (*r* = −.13, *p* < .05). Moreover, eco-anxiety positively correlated with avoidant coping (*r* = .54, *p* < .01) and negatively correlated with problem-focused coping (*r* = −.14, *p* < .01) and emotion-focused coping (*r* = −.17, *p* < .05). Problem-focused coping positively correlated with emotion-focused coping (*r* = .70, *p* < .01) and negatively correlated with avoidant-focused coping (*r* = −.41, *p* < .01). Finally, emotion-focused coping negatively correlated with avoidant-focused coping (*r* = −.43, *p* < .01).

**Table 2.** Correlations among study variables (*N* = 554)

Measures	1	2	3	4	5	6
1. Post-traumatic stress	1	.11*	.64**	−.17*	−.19*	.52**
2. Environmental degradation		1	.14*	−.15*	−.13*	.40**
3. Eco-anxiety			1	−.14*	−.17*	.54**
4. Problem-focused coping				1	.70**	−.41**
5. Emotion-focused coping					1	−.43**
6. Avoidant-focused coping						1

** significant at (α ≤ .01) * si gnificant at (α ≤ .01)

### Structural equation modeling

**Figure 2.**
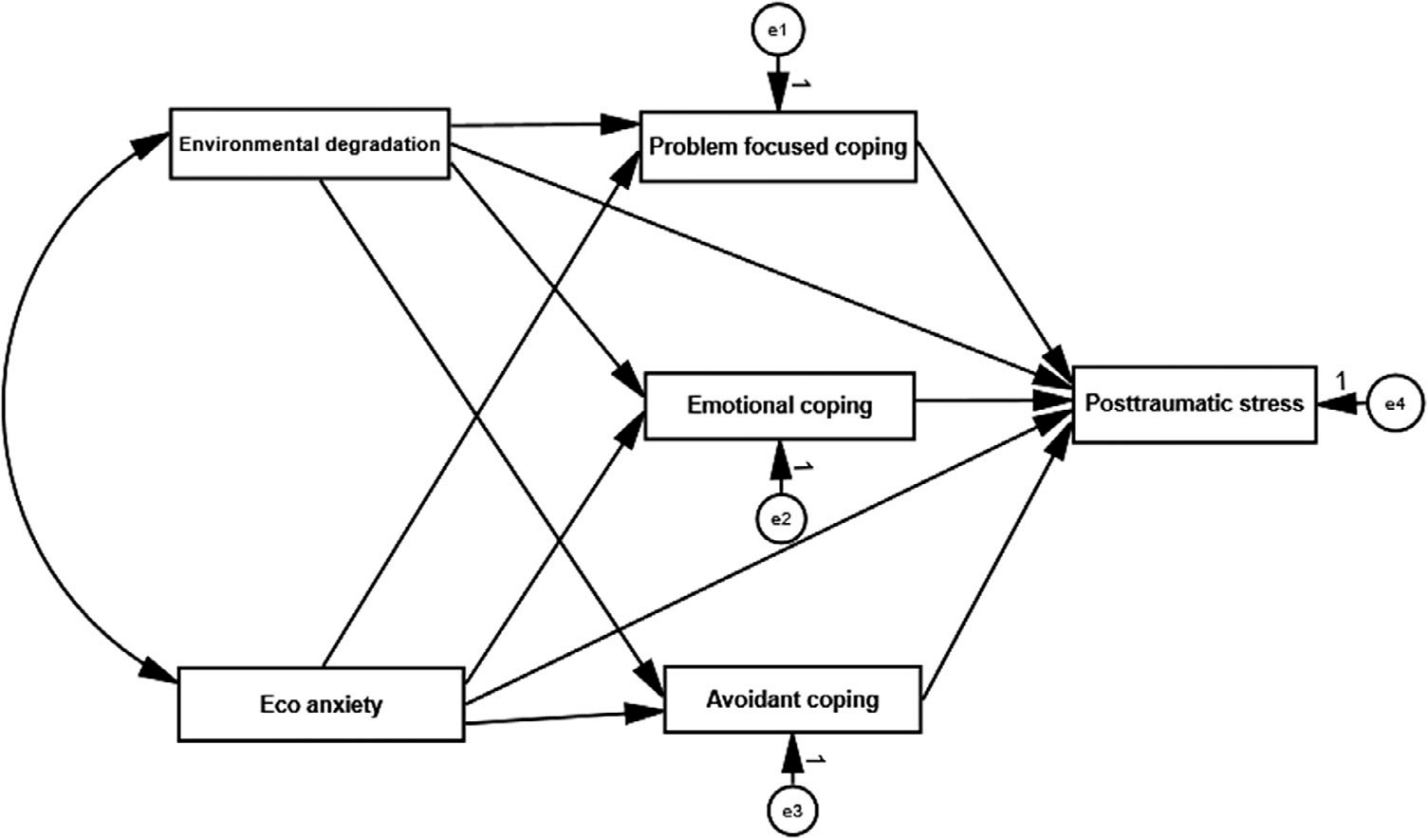
Structural equation modeling for environmental degradation and eco-anxiety on post-traumatic stress, and the mediating roles of coping strategies.

SEM results are shown in Figure 2, with environmental degradation and eco-anxiety as predictors, problem-focused coping, emotion-focused coping and avoidant-focused coping as mediating variables, and post-traumatic stress as an outcome variable. Our study showed that problem-focused coping, emotion-focused coping and avoidant-focused coping mediated the association between environmental degradation, eco-anxiety and post-traumatic stress. The model showed good fit indicators, as all paths were significant (*χ* ^2^_(11)_ = 224.74; *p* = .001; GFI= .96; AGFI= .97; RMSEA= .03; NFI= .96; CFI= .97).

Concerning the mediating hypothesis (H3), our model revealed a standardized total effect of eco-anxiety on PTSSs (βX, M = .29; *p* < .001), and a standardized total effect of environmental degradation on PTSSs (βX, M = .27; *p < .001*). Moreover, the model revealed a standardized total effect of behavioral coping on PTSSs (βX, M = −14; *p < .001*) and a standardized total effect of emotional coping on PTSSs (βX, M = −11; *p < .001*); a standardized total effect of avoidant coping on PTSSs was also noted (βX, M = 43; *p < .001*). Moreover, the model showed a standardized total effect of eco-anxiety on PTSSs (βX,M, Y = . 29; *p < .01*), with significant indirect effect via behavioral coping (βX,M, Y = −. 16; *p < .05*), emotional coping (βX,M, Y = −.19; *p < .01*) and avoidant coping (βX,M, Y = . 14; *p < .01*) . Moreover the model showed a standardized total effect of environmental degradation on PTSSs (βX,M, Y = . 27; *p < .01*), with significant indirect effect via behavioral coping (βX,M, Y = −. 14; *p < .05*), emotional coping (βX,M, Y = −. 16; *p < .01*) and avoidant coping (βX,M, Y = .17; *p < .01*).

## Discussion

The present study sought to explore the effect of environmental degradation and eco-anxiety on people’s post-traumatic reactions in an environment characterized by political instability and violence such as the West Bank of Palestine. Coping strategies have finally been tested and confirmed to have a mediating role. People’s quality of life in Palestinian territories is affected by the military occupation and lack of infrastructure, causing harm to the local ecosystem and their psychological well-being (Isaac and Hilal, 2011).

The results of this study provide valuable insights into the complex interplay between environmental degradation, eco-anxiety, coping strategies and post-traumatic stress symptoms among Palestinian adults experiencing ongoing military violence and loss of freedom. The findings support the study hypotheses and shed light on the nuanced relationships within this context.

First, the descriptive statistics revealed that environmental degradation was notably pronounced among the participants who are describing territories severely affected by the ongoing occupation. This underscores the environmental challenges faced by Palestinians living in the West Bank. Post-traumatic stress symptoms, eco-anxiety and coping strategies, on the other hand, exhibited moderate scores. These findings suggest that while environmental concerns and psychological distress are present, they may not be overwhelmingly pervasive in this population that might show sufficient coping strategies to deal with a deteriorated living environment. Furthermore, the high reliability of the measures employed in this study adds robustness to the results and underscores the validity of the data.

The correlational analysis yielded several significant associations among the study variables, illuminating the complex web of relationships between environmental issues and post-traumatic reactions in this context (Berger, 2015). Notably, post-traumatic stress symptoms were positively correlated with environmental degradation, eco-anxiety and avoidant coping, suggesting that individuals experiencing higher levels of environmental degradation and eco-anxiety may be more prone to post-traumatic stress symptoms widespread among the civilian population living under occupation. Conversely, post-traumatic stress symptoms displayed negative correlations with problem-focused coping and emotion-focused coping, indicating that individuals employing these coping strategies may experience fewer post-traumatic stress symptoms.

Environmental degradation exhibited a positive correlation with eco-anxiety and avoidant coping, aligning with the notion that individuals facing environmental challenges may experience heightened anxiety and may resort to avoidance as a coping mechanism. Moreover, the negative correlations between environmental degradation and problem-focused coping, as well as emotion-focused coping, suggest that individuals experiencing environmental degradation may be less likely to employ proactive or emotionally expressive coping strategies.

Eco-anxiety displayed a positive correlation with avoidant coping and negative correlations with problem-focused coping and emotion-focused coping. This suggests that individuals with higher levels of eco-anxiety may tend to avoid confronting environmental concerns directly and may struggle with active problem-solving and emotional expression in response to these concerns.

### Structural equation modeling

The SEM analysis provided a comprehensive understanding of the relationships between the study variables. The model fit indices indicated a strong fit, bolstering the validity of the proposed model. The mediating role of coping strategies in the relationships between environmental degradation, eco-anxiety and post-traumatic stress symptoms is a noteworthy finding.

The standardized total effects revealed that both environmental degradation and eco-anxiety had direct and positive associations with post-traumatic stress symptoms. This implies that individuals experiencing higher levels of environmental degradation and eco-anxiety are more likely to exhibit post-traumatic stress symptoms.

Furthermore, the mediating hypothesis (H3) was supported. Coping strategies, including problem-focused coping, emotion-focused coping and avoidant coping, played significant mediating roles in the associations between environmental degradation, eco-anxiety and post-traumatic stress symptoms. These findings highlight the importance of coping strategies in mitigating or exacerbating the psychological impact of environmental challenges.

### Implications

These results have several implications for research and practice. First, they underscore the importance of considering both environmental factors and psychological well-being in the context of environmental degradation. Environmental policies and interventions should not only focus on mitigating environmental harm but also on addressing the potential psychological consequences.

Second, the findings emphasize the need for tailored psychological support and coping strategies for individuals experiencing eco-anxiety and post-traumatic stress symptoms in the face of environmental challenges. Interventions that promote adaptive coping mechanisms, such as problem-focused and emotion-focused coping, may be particularly beneficial.

Third, mental health interventions in Palestine need to be supported through advanced training and capacity building for mental healthcare professionals, as well as through supporting the academic programs by bringing in people with high qualifications in mental health, in addition to linking those programs to the international community.

Lastly, these findings contribute to the growing body of literature on eco-anxiety and its relevance in regions facing significant environmental degradation. Recognizing the psychological impact of environmental issues can inform comprehensive approaches to environmental sustainability and mental health.

### Limitations

While this study provides valuable insights, it is not without limitations. The cross-sectional nature of the study limits causal interpretations. Longitudinal studies could provide a better understanding of the temporal relationships between variables. Additionally, the study focused on a specific population (Palestinian adults in the West Bank), which may limit the generalizability of the findings to other contexts. Studies targeting other regions of Palestine, such as East Jerusalem and the Gaza Strip, are needed. Also, other groups affected by environmental degradation and conflict, such as Palestinian refugees and victims of political violence, should be studied to generalize the findings. Moreover, our study only tested the mediating role of coping strategies in the relationship between environmental degradation, eco-anxiety and PTSD. Exploring potential moderating or mediating factors that may influence these relationships, such as social support, cultural factors or exposure to trauma, should be considered in future studies. Finally, the current study used self-reports to test the association between environmental degradation, eco-anxiety and PTSD, and whether coping strategies mediate the association between these variables. To enhance the generalizability and validity of findings, future research could benefit from a combination of qualitative and quantitative methods.

In conclusion, this study contributes to the understanding of how environmental degradation, eco-anxiety, coping strategies and post-traumatic stress symptoms intersect. It highlights the complex relationships among these variables and underscores the importance of addressing both environmental and psychological well-being in regions facing environmental challenges.

## Data Availability

The datasets generated during and/or analyzed during the current study are available from the corresponding author on reasonable request. The datasets generated during and/or analyzed during the current study are available in the OSF repository (https://osf.io/xw87t/files/osfstorage).
