# Supplementary material for: Environmental degradation, eco-anxiety and post-traumatic stress symptoms among Palestinian adults: The mediating role of coping strategies
Source: Glob Ment Health (Camb). 2024 Oct 8;11:e105. doi: 10.1017/gmh.2024.40 (PMC11588413; doi:10.1017/gmh.2024.40)
Supplement: Peer review history

# Editor report: Recommendation: Environmental degradation, eco-anxiety and post-traumatic stress symptoms among Palestinian adults: The mediating role of coping strategies — R0/PR2

**DOI:** 10.1017/gmh.2024.40.pr2

**Affiliations:** Faculty of Business, Technology, and Education , Ilia State University, Georgia

No accompanying comment.

# Editor report: Decision: Environmental degradation, eco-anxiety and post-traumatic stress symptoms among Palestinian adults: The mediating role of coping strategies — R0/PR3

**DOI:** 10.1017/gmh.2024.40.pr3

**Affiliations:** Johns Hopkins University Bloomberg School of Public Health, United States

No accompanying comment.

# Editor report: Recommendation: Environmental degradation, eco-anxiety and post-traumatic stress symptoms among Palestinian adults: The mediating role of coping strategies — R1/PR5

**DOI:** 10.1017/gmh.2024.40.pr5

**Affiliations:** Faculty of Business, Technology, and Education , Ilia State University, Georgia

No accompanying comment.

# Editor report: Decision: Environmental degradation, eco-anxiety and post-traumatic stress symptoms among Palestinian adults: The mediating role of coping strategies — R1/PR6

**DOI:** 10.1017/gmh.2024.40.pr6

**Affiliations:** Johns Hopkins University Bloomberg School of Public Health, United States

No accompanying comment.
